# Unusual Cases of Syncope in the Pediatric Age Group

**DOI:** 10.1155/2021/8849766

**Published:** 2021-03-12

**Authors:** Riti Bhalla, Chantal Soobhanath, Sarah Celebi, Manoj Chhabra, Pramod Narula

**Affiliations:** ^1^St. George's University, School of Medicine, St. George, West Indies, Grenada; ^2^Department of Pediatrics, New York Presbyterian Brooklyn Methodist Hospital, 506 6th St, Brooklyn, New York 11215, USA

## Abstract

Syncope is common in the pediatric population and occurs in up to 15 percent of children prior to the end of adolescence. While the etiology of syncope in children is often benign and the majority of cases can be explained by isolated changes in vasomotor tone, a thorough evaluation is warranted to rule out more serious, life-threatening causes of syncope. Here, we present three atypical cases of syncope: a young judo player with recurrent syncope and dizziness, a teenage boy with syncopal episodes always preceded by stretching, and a child who experienced urticaria before losing consciousness. Herein, we review the pathophysiology, diagnosis, and management of syncope in children and adolescents.

## 1. Introduction

Syncope is characterized by a sudden, brief, and self-limited episode of loss of consciousness and inability to maintain postural tone. It is a common clinical problem that accounts for an increasing number of outpatient visits and prompts further evaluation by primary care providers and pediatric cardiologists [1]. Though syncope is often benign and can be attributed to vasovagal changes leading to transient cerebral hypoperfusion, cardiac-mediated causes, including arrhythmias and structural heart disease, need to be ruled out. Herein, we sought to report less common etiologies of syncope, in order to avoid misdiagnosis and emphasize the importance of thorough history-taking in evaluating syncope.

## 2. Case 1

A 10-year-old female presented to a cardiology clinic for the evaluation of recurrent episodes of exertional and nonexertional dizziness. She is a competitive judo player and has occasionally reported lightheadedness after 30 minutes of participation in judo and while running. Additionally, she endorsed frequent palpitations during exercise which last for a few minutes before gradually subsiding. She has had two syncopal episodes in the past, one of which occurred while standing and another during judo practice. Upon further questioning, it was disclosed that the patient had been following a regimen of extreme fluid restriction several days prior to the competition to maintain her weight class. Recommended workup included electrocardiogram (ECG), transthoracic echocardiogram, and exercise stress test. ECG and transthoracic echocardiogram were both normal. A treadmill stress test was performed, in which the patient was able to exercise for 9 minutes and 58 seconds, but it was terminated early due to the patient's complaint of dizziness. Heart rate ranged from baseline of 75 beats per minute (bpm) to a maximum of 193 bpm. Baseline blood pressure was 113/54 mmHg and progressively dropped to a low of 94/50 mmHg nine minutes into the test, rising to 107/59 mmHg with recovery from exercise. When the stress test was performed, the patient had been on a fluid restriction regimen days prior to preparation for the competition. She was advised to increase her fluid and salt intake. The patient was also instructed to refrain from exercise for the first two weeks of wearing the event monitor and resume exercise for the following two weeks to monitor exertional and nonexertional syncope. She has been asymptomatic following increase in fluid intake.

## 3. Case 2

A 17-year-old male presented to a cardiology clinic for the evaluation of syncope. He had been having syncopal episodes approximately once per month for the past two years, and these episodes had recently increased in frequency. According to the patient's mother, the episodes were always preceded by arm stretching (e.g., while reaching for something from the closet), and the patient endorsed holding his breath while stretching. During the most recent episode, he was stretching, became dizzy, and subsequently lost consciousness, followed by a brief convulsion of the upper extremities. The patient denied a history of palpitations, shortness of breath, or chest pain. His family history was significant for migraines in his sister. Cardiac evaluation was unremarkable with normal ECG and echocardiogram. The patient was discharged with the impression of vasovagal syncope caused by unintentional Valsalva maneuver during stretching, and a subsequent tilt-table test was negative.

## 4. Case 3

A 13-year-old female with a past medical history of asthma and eczema presented to clinic with a complaint of syncopal episodes, in which she would experience generalized exercise-induced urticaria prior to losing consciousness. A few hours before the episodes, she also complained of flushing, dizziness, rhinorrhea, and shortness of breath. Per the patient's mother, she had never had an allergy test performed but was suspected to be allergic to pet dander and had recently been exposed to a dog. As part of her workup, an ECG, echocardiogram, cardiac computerized tomography (CT), cardiac magnetic resonance imaging (MRI), and a treadmill stress test were performed. The ECG, echocardiogram, CT, and MRI were all within normal limits. The stress test lasted for 11 minutes, with a blood pressure of 142/68 mmHg and heart rate of 171 bpm ten minutes into exercise, and a subsequent drop to a blood pressure of 120/68 mmHg and heart rate of 200 bpm. The stress test was discontinued due to the patient experiencing dyspnea, chest tightness, and generalized urticaria. She was started on antihistamines with no recurrence of the episodes.

## 5. Discussion

Syncope is most often vasovagal in nature, which is typically benign. It can be precipitated by emotional or orthostatic stress and is associated with prodromal symptoms such as lightheadedness and fatigue [2, 3]. The workup for syncope may involve a baseline ECG, stress test, telemetry, transthoracic echocardiogram, and tilt-table test [4−6]. Dietary salt supplements and mineralocorticoids (e.g., fludrocortisone) have been found to relieve syncopal symptoms as they increase extracellular fluid volume, thereby improving orthostatic tolerance. At times, patients have been treated with *β*-blockers, scopolamine, theophylline, and serotonin reuptake inhibitors, with variable results [7, 8].

In Case 1, we described a case of syncope in a young judo competitor which resulted from forceful fluid restriction to maintain weight class. Not only did her decreased fluid intake reduce her plasma volume, but she also had increased insensible loss secondary to exercise. Moreover, exercise can cause vasodilation to the exercising muscles, which in turn can reduce blood pressure if there is insufficient vascular volume [9].

Case 2 described a patient with multiple episodes of syncope following stretching. “Stretch syncope” is a rarely reported cause of syncope in the pediatric population. Although its pathophysiologic mechanism is not fully understood, it is thought to occur due to an unintentional Valsalva maneuver, which indicates adrenergic dysfunction. However, there is conflicting evidence regarding the cardiovascular responses during Valsalva maneuver preceding stretch syncope, and further studies are needed to elucidate its underlying mechanism [10].

Case 3 presented a case of syncopal episodes preceded by exercise-induced urticaria and allergic rhinitis. We propose that these episodes were related to exercise-mediated histamine release. Mast cell degranulation with release of mediators, such as histamine, causes vasodilation, which results in reduced venous return leading to syncopal episodes and vasodilatory shock, in rare cases. While there are reports of several prophylactic treatments used to prevent anaphylactic episodes, there remains lack of adequate evidence to support the use of any specific regimen [11]. Exercise-induced anaphylaxis is typically associated with a cofactor triggering the anaphylaxis, such as a food allergy or exercise in a warm or cool environment. Management is similar to other causes of anaphylaxis, including administration of intramuscular epinephrine, prescription of EpiPen for the patient to always have on hand, and education for patients and families on recognizing symptoms, acute management, and seeking medical attention [11].

We present these atypical cases to promote awareness of the less common causes of syncope and to emphasize the importance of detailed history-taking in the diagnosis of syncope. Although the overarching cause of syncope is vasovagal in nature, it is important for the medical professional to be aware of the unusual causes so as to ensure efficient diagnosis and effective treatment.
